# Intravenous bisphosphonates for postmenopausal osteoporosis

**Published:** 2010

**Authors:** Peyman Mottaghi

**Affiliations:** a Assistant Professor, Department of Internal Medicine, Faculty of Medicine, Isfahan University of Medical Sciences, Isfahan, Iran. E-mail: motaghi@med.mui.ac.ir

**Keywords:** Bisphosphonates, Postmenopausal, Osteoporosis, Pamidronate, Ibandronate, Zoledronic Acid

## Abstract

Numerous clinical studies have shown bisphoshonates (BPs) to be useful and cost-effective options for the fractures prevention and postmenopausal bone loss. The use of oral bisphoshonates is an established option for managment of osteoporosis in postmenopausal women, but many of them complaint from gastrointestinal side effect or frequently dosed oral regimens. To improve upon the suboptimal therapeutic compliance in postmenopausal women, newer, longer-acting intravenous formulations of BPs has been approved for intermittent administration in postmenopausal women. These preparations would become an option for patients who can not tolerate oral BPs or it was ineffective in increasing their bone density.

This article proposed to review effectiveness and tolerability of intravenous BPs in postmenopausal women with osteoporosis.

Bisphosphonates (BPs), which are potent bone resorption inhibitors, now are the mainstay of prevention and treatment for women with bone loss. These drugs are often used as first treatment option for osteoporosis since 1960s when the first BPs was developed as drugs for humans.^1^,^2^ Despite efficacy of BPs near 50% of patients discontinue using their prescribed drug within the first year or continue it inappropriately.^3^ Poor compliance is associated with negative outcomes, including increased fracture risk. Tolerability and safety are among the causes of poor compliance.^4^,^5^ Intravenous bisphosphonates avoids the gastrointestial intolerance and the complex dosing instruction of the oral route ensuring full compliance which may provide improved efficacy.^6^,^7^ However, there are some concerns regarding potent intravenous bisphosphonates including: zoledronic acid, ibandronate and pamidronate with respect to tolerability, mainly the acute phase response and to safety, mainly a theoretical risk of over suppression of bone turnover, renal toxicity and osteonecrosis of the jaw.^6^,^8^

To review bone loss prevention, safety, tolerability and dosing regimens of intravenous formulation of bisphosphonates PubMed/MEDLINE, ISI Web of Science and Springer data bases were searched for English-language articles from 1991 to 2009.

## 

### Mechanism of Action and Pharmacology

Bisphosphonates are analogs of pyrophosphate; an endogenous inhibitor of bone resorption that during the process of bone resorption and after binding to bone mineral are taken up by osteoclasts.^9^–^11^ After binding to mineralized bone surface, BPs are taken up from surrounding fluid by osteoclasts. Once inside the cell, they inhibit bone resorption by decreasing solubility of bone substance and changing into mineralization because of their incorporation into hydroxyapatite crystals and into bone matrix.^12^ Current evidence supports the following effects of BPs on osteoclasts: reduction of the lifespan by enhancement of apoptosis; and inhibition of osteoclast formation, recruitment and activation that accompanied by lowering activity of mature osteoclasts.^1^,^9^,^11^ These effects ultimately lead to suppression of bone turnover and longer life span for each remodelling unit, which in turn permits more complete secondary mineralization of each resorption pit and increased bone mass. The result of this process is reduction in the risk of fractures.^3^,^6^,^9^

Although, the action of all BPs appear to be similar, but various BPs display some differences in their affinity for bone resorption sites and have specific properties of the structure.^12^ They are categorized into generations based on side chain additions and antiresorptive potency. Non-nitrogen containing BPs are converted to nonhydrolyzable cytotoxic analogs of adenosine triphosphate (ATP) intracellular and intracellular accumulation of these analogs interferes with osteoclast function and results in osteoclast apoptosis.^9^,^10^

The more potent nitrogen-containing BPs act by either causing direct toxic effects on osteoclasts or inducing osteoclast apoptosis via interference with intracellular signalling functions of key regulatory proteins.^13^ Furthermore, histological evidence suggests that BPs may alter the balance between bone formation and resorption by stimulating the proliferation of preosteoblast cells and increasing osteoblast production of osteoprotegerin, an antiresorptive protein.^10^,^13^ This would allow an increase in bone mineral density (BMD) as a result of slowed remodelling and increased mineralization.

### Pharmacodynamics

BPs are synthetic compounds which are absorbed into the body, stored in bones, and eventually excreted unchanged. Interactions with other pharmaceutical agents have not been observed. BPs can be administered orally, intravenously, intranasaly or transdermaly, but these last two modes have been abandoned because of irritation of mucus membrane and skin.^10^,^14^ The intestinal absorption of BPs is minimal and varies from less than 1% to 10%; its dose dependent and decreased when ingested simultaneously with food, especially if rich in calcium such as milk products, or with coffee and orange juice.^6^ When given intravenously more than 70% of total dose reach to bone and 20-50% is deposited on the bone surface and the rest is excreted in urine or feces within 24 hours.^15^ Although the total dose is important for the effect on bone resorption, the uptake on bone resorption surfaces appears to be the major determinant of the antiresorptive effect.^16^

Because of their rapid uptake by bone, soft tissues and internal organs are only briefly exposed to BPs. They remain in the skeleton for many years and this prolonged attachment explains their extended duration of action.^17^ There is no evidence that BPs deposited in the bone retains any pharmacologic activity or exert any harmful effects on the quality of the bone involved. The dosage and half-life of the BPs given to patients with renal insufficiency or on hemodialysis must be carefully calculated for each individual patient.^3^

### Efficacy of Intravenous BPs in Postmenopausal Osteoprosis

Fracture prevention and treatment of bone loss in postmenopausal women often requires prolonged pharmacologic treatment.^18^,^19^ Epidemiologic analyses demonstrate that as many as 25% of patients fail to follow the oral dosing instructions, or continue the medication one year after it is prescribed.^20^,^21^ Post-marketing studies of oral BPs showed that up to 38% of patients experience gastrointestinal problems^22^,^23^ and hence as many as 19% of patients discontinue follow the oral instruction.^20^

Intravenous therapy has gained a high degree of compliance, especially with patients who are already taking a number of other drugs. Additional advances are 100% bioavailability and no gastrointestinal side effects. Moreover, the effect on bone density and fracture rate are comparable to dose of oral therapy.^15^ Therefore, intravenous BPs formulations by long dosing interval and infrequent upper digestive tract adverse events, appear a logical alternative to oral preparations.

The highest rate of increase in bone density occurs during the first 12 months and the optimal duration of bisphosphonate therapy is 1-3 years, depending on the severity of osteoporosis and the increase in bone density. After 3 years of treatment, results of annual measurements of BMD will determine when BPs therapy should be resumed.^3^,^18^

### Pamidronate

Currently pamidronate has been used as an off-label indication for patients who are intolerant to, or have contraindication for oral BPs.^8^,^24^

In a retrospective analysis of monthly intravenous pamidronate (60 mg) and daily oral alendronate (various doses), no difference in the development of new vertebral fractures observed between the groups.^25^ This study indicates that for treatment of postmenopausal osteoporosis, intravenous pamidronate is at least as good as oral alendronate. Based on retrospective data, intravenous pamidronate and oral alendronate (various doses) increased lumbar spine BMD significantly after 3 years of treatment.^25^,^26^

Small but placebo-controlled trials are available that highly suggest beneficial effect of pamidronate on bone density.^27^,^28^ A prospective study compared the affects of 10 mg/d of oral alendronate and 60 mg/3months of intravenous pamidronate therapy for one year showed that the bone density in the lumbar spine significantly increased in the groups (about 4.0% vs. baseline, in both the alendronate and the pamidronate groups). Measurements of the bone density in total hip also showed significant increase in both the pamidronate and the alendronate groups (3.3% and 2.9% vs. baseline, respectively).^29^ They conclude that treatment with intravenous pamidronate every 3 months appears to be as effective as daily oral alendronate and for those who cannot tolerate an oral bisphosphonate, intravenous pamidronate is an acceptable alternative to oral bisphosphonates.^29^

A clinical trial that used pamidronate 30 mg intravenously every 3 months resulted in 6% increase in bone density of the femoral neck and 10% at the lumbar spine after 2 years administration in postmenopausal women with osteopoeosis.^28^ Comparable increases in proximal femur and vertebral bone density were obtained with either oral alendronate or intravenous pamidronate.^29^ A single 90 mg intravenous dose of pamidronate was found to prevent bone loss after hip arthroplasty.^30^ Currently intravenous pamidronate has no approval for use in postmenopausal osteoporosis but it has been used to treat osteoporosis in patients who were not able to tolerate oral BPs.^24^

### Ibandronate

The third bisphosphonate to achieve FDA approval was Ibandronate. In animal models, it was 2 to 10 times more potent than alendronate.^31^,^32^ Efficacy of ibandronate, administered as oral daily or intermittently with interval dose of more than one month for osteoporosis of postmenopausal women, was shown in various studies.^33^–^35^ Due to associated gastrointestinal side effects of daily oral BPs, ibandronate is also available as an intravenous formulation.

Results of a recent study showed that treatment of postmenopausal osteoporosis with both oral and intravenous ibandronate (every 3 months) reduces bone resorption markers and significantly increases bone density.^36^

A recent multicenter, double-blind, placebocontrolled study assessed the safety and efficacy of various intermittent intravenous doses of ibandronate (0.5 mg, 1 mg, and 2 mg) in 629 postmenopausal women for 1 year.^37^ This study showed that intravenous ibandronate therapy produced a dose-dependent increase at the lumbar spine bone density up to 2.5%, while a 0.4% decline observed with the placebo. Additionally, the 2 mg intravenous of ibandronate every three months produced marked increases in bone density of 2.1%, 1.7%, and 0.9%, at the trochanter, total hip and femoral neck area, respectively.^37^ In another study, intermittent treatment with the 2 mg IV ibandronate resulted comparable annual increment of bone density with 5 mg oral alendronate and risedronate.^38^

Two dose regimen of the intravenous formulation (2 mg every 2 months, 3 mg every 3 months) and 2.5 mg daily oral dose of ibandronate were compared in a double-blind trial on 1395 postmenopausal women. After 1 year, lumbar spine bone density increased 3.8% in the oral 2.5 mg daily group, 4.1% in the 3 mg/3 months group and 5.1% in the 2 mg/2months group. Similarly, superior results were also observed in both intravenous groups for increase in proximal femoral and total hip bone density compared to oral treatment (p < 0.001).^36^

Two trials studied the efficacy of various doses of intravenous ibandronate administered every 3 months for the treatment of postmenopausal osteoporosis. Results demonstrated an average increase in bone density at the lumbar spine of 2.5 to 3.5% over placebo, and statistically significant increases of 4.2% at the trochanter and 2.9% at total hip bone density, after 12 months of therapy. Both intravenous and oral ibandronate significantly increased bone density in postmenopausal women.^38^,^39^ For evaluation of the efficacy and safety of intravenous ibandronate in the Dosing IntraVenous Administration (DIVA) study, administration of ibandronate every 2 months or every 3 months was compared with approved daily oral 2.5 mg ibandronate regimen. In this report, the presented results showed that both intravenous regimens increased bone density in lumbar spine from baseline (5.1% and 4.8%) after one year of treatment; that was greater than that provided by daily oral ibandronate regimen (3.8%).^38^

According to the DIVA study, for post-menopausal women with osteoporosis, ibandronate injections every 2 or 3 months are at least as effective and similarly well tolerated as daily oral ibandronate.

### Zoledronic Acid

It is the newest and most potent nitrogen-containing BPS to date and available only as an intravenous preparation (Table 1).^40^ Only small doses of zoledronic acid are required for inhibition of bone resorption and long dosing intervals can enhance adherence, especially in patients unable to comply with oral dosing, owing to severe co-morbidities or GI intolerance.^8^ Two large placebo-controlled randomized trials revealed effectiveness of zoledronic acid in increase of bone density and reduce of hip and vertebral fractures.^41^,^42^ In a study, with different doses and intervals, zoledronic acid dose-dependently increased bone density in both femoral neck and lumbar spine with all regimens. All patients in the zoledronic acid groups, including a 4 mg dose given once during the 12 month study, experienced increase in bone density at the lumbar spine. The increase at the femoral neck (3.1-3.5%) and lumbar spine (4.3-5.1%) bone density achieved with zoledronic acid were significantly higher than placebo group, but no differences were noted between the zoledronic acid groups. The results for total body bone density were similar between all zoledronic acid groups and placebo.^43^ Remarkably, sustained suppression of bone resorption, accompanied by increased bone density, was obtained in up to one year even with a single 4 mg dose.^43^ Biochemical markers of bone resorption remained suppressed for the length of the study, whereas no changes were seen in the placebo group. Because the bone resorption markers were still suppressed at 12 months, a dosing interval longer than 12 months may also be effective, but the maximum dosing interval is unknown.^43^

**Table 1:** Intravenous bisphosphonate regimens for treatment of postmenopausal osteoporosis

Substance	Approved dosage*	Trade names	FDA ap proval†
Pamidronate	30-60 mg infusion every 3 months	Aredia®	Not approved
Ibandronate	2 mg injection every 3 months	Bonadronat^®^ Boniva^®^ Zometa^®^	Prevention and treatment
Zoledronate	2 mg infusion every 3 months or 5 mg annually	Acasta^®^, Reclast^®^	Prevention and treatment

* Data adapted from references number 66-68

† FDA: Center for Drug Evaluation and Research [homepage on the Internet]. Accessed June 28, 2009. Available at: http://www.accessdata.fda.gov/scripts/cder/drugsatfda

A recent placebo-controlled, double-blind trial of annual zoledronic acid for 3 years on 7736 postmenopausal women with osteoporosis resulted in a 41% and 70% reduction in risk of hip and vertebral fracture, respectively.^44^

Findings of a large-scale, randomized, controlled trial for antifracture efficacy of intravenous zoledronate at the dose of 5 mg in post-menopausal women with osteoporosis have led to speculation that zoledronic acid may be used as prophylactic drug for postmenopausal osteoporosis.^45^,^46^ It is possible that, after three annual infusions, there is enough zoledronic acid in the bone to be re-cycled, allowing maintenance of BMD and risk reduction.^47^

### Safety and Tolerability of Intravenous BPs

The bisphoshonates are well tolerated but before initiation of intravenous therapy, complete blood count, kidney and liver function tests, calcium, phosphate and alkaline phosphates in the serum must be checked. Contraindications for bisphosphonates include hypersensitivity to the drug and low-serum calcium; these agents should not be used in women who are pregnant or nursing.^48^ They also should not be given together with aminoglycosides and use with cautious in severe renal dysfunction (creatinine clearance < 35 mL/min).^48^

Fractures or orthopaedic prostheses do not constitute contraindications to intravenous bisphosphonate therapy, on the contrary, callus formation is increased and fractures are repaired more rapidly under bisphosphonate therapy.^49^

When bisphoshonates are administered intravenously, because of transient drop in the serum calcium concentration, it is important to monitor the rate of infusion and clinical relevant hypocalcaemia must be observed after rapid infusion of high doses or with concomitant administration with aminoglycosides.^48^

The most frequently reported treatment-related adverse events involved the musculoskeletal and GI systems, consisting primarily of upper abdominal pain (3.0-3.6%), dyspepsia (3.4-4.1%), flu-like illness (0.9-4.1%) and arthralgia (2.4-3.6%).^38^,^50^

Flu-like syndrome reported with the use of intravenous BPs is common, but this is usually mild, short-lived, self-limiting and often occurs only after the first dose.^43^,^50^,^51^ This acute phase reaction which begins the day after BPs infusion has been seen in 20-40% of all patients consist of fever, headache, bone and joint pains and fatigue. These reactions lasts only 1 to 2 days and do not leave any long term side effects and occurs only after the first infusion, rarely after the second and then is very mild.^43^

A safety profile similar to placebo has demonstrated with intravenous ibandronate and no evidence of renal toxicity at doses of 0.5, 1.0, or 2.0 mg given at 3 month intervals.^52^ A serious concern with the use of zoledronic acid is the potential for acute renal failure or renal function impairment due to formation of insoluble complexes in the blood with infusion of BPs.^53^ Renal impairment is most likely related to high-dose use and rate of infusion.^54^ Consequently, intravenous administration of BPs should be slow and considerably diluted. No evidence of renal function impairment with annual (4 mg), biannual (2 mg), and quarterly (0.25, 0.5, 1 mg) use of zoledronic acid infusions find in postmenopausal women with osteoporosis.^43^

Renal insufficiency per se is not a contraindication for bisphosphonates.^3^,^55^ Administration of bisphosponates is not limited by renal insufficiency but they should only be given to patients with creatinine clearance of more than 30-35 mL/min or with serum creatinine concentrations less than 2.0 mg/dL.^55^ There are few data on the effect or safety of bisphosphonates in patients with GFR < 30-35 mL/min. Nevertheless, bisphosphonate use is a worthy consideration in high-risk patients with chronic sever renal insufficiency after evaluating the bone histomorphometry to exclude other forms of renal osteodystrophy that may mimic osteoporosis before use of any bisphosphonate.^56^

It is also advisable to reduce the dose and prolong the infusion time in patients with renal insufficiency. In patients on haemodialysis, the dose should be reduced by 25% and the different half lives of BPs must be taken into account (ibandronate, zoledronate 1 hour, and pamidronate 10-16 hours).^57^ The potential renal damage that can be seen with rapid infusions of zoledronic acid is rare with longer than 15 minutes infusion rates and it is safe even in patients with pre-existing diabetes and hypertension or on non-steroidal anti-inflammatory drugs (NSAIDs).^58^ Intravenous ibandronate injections have not been associated with renal failure in the populations studied, thus it might be safe to use in patients with renal insufficiency.^57^

Osteonecrosis of the jaw (ONJ) is an area of exposed nonhealing bone due to breakdown of localized areas of the jaw bone. Although ONJ is frequently a painful condition, but about one-third of patients do not experience pain.^59^ It was rarely reported before the advent of bisphosphonates therapy and previously was seen in patients who had received radiation therapy to the jaw, but it can be a serious adverse effect in patients taking BPs. Although most of cases occur after dental extraction, it also closely related to dental infection and trauma (such as dental surgery) but can also occur spontaneously.^59^ Most reports on ONJ are about patients with multiple myeloma or breast cancer. The patients who have developed the condition have received intravenous BPs at high doses, several times more than for osteoporosis, and with frequent intervals (such as monthly).^59^,^60^ Most of cases have had dental pathology or oral surgery and received chemotherapy, corticosteroids or radiation therapy.^60^,^61^ The overall incidence of ONJ in patients receiving BPs for osteoporosis is very low and the level of evidence for an association between ONJ and the use of intravenous BP for the treatment of osteoporosis remains weak.^59^

A few number of atrial fibrillation events seen in the zoledronic acid trials for post-menopausal osteoporosis and a prior history of cardiac arrhythmias was an important risk factor for it.^41^,^43^ To date FDA has not considered these events as necessarily being directly related to intravenous zoledronic acid and it remains to be determined if history of prior cardiac arrhythmia should be a precaution in considering bisphosphonate use. Because of reported cardiac irregularities such as atrial fibrillation, one of special precautions for use of intravenous BPs is elderly patients, but these paroxysmal disturbances in cardiac rhythm often mild and subsides by temporary discontinuation of infusion.^62^

In patients who are hypocalcemic or deficient in vitamin D, use of intravenous bisphosphonates can rapidly worsen hypocalcaemia and it is extremely important to identify and correct disorders of bone metabolism before intravenous administration of these compounds.^15^ Intravenous therapy with BPs also can worsen untreated subclinical vitamin D deficiency, leading to osteomalacia and increased risk of stress fractures.

Therefore, all patients candidate to use intravenous preparations of BPs must receive adequate amounts of vitamin D and calcium by diet or supplements before institution of the drug.

Intravenous administration of BPs may cause anorexia, nausea, vomiting, and electrolyte abnormalities.^50^ Less commonly, the intravenous route may cause hypertension and decreased renal function.^50^ BPs, regardless of route of administration, have also been associated with central nervous system adverse events, such as hallucinations (auditory and olfactory) and ocular disturbances (conjunctivitis, blurred vision, eye pain, and inflammation). Although rare in occurrence, these side effects can lead to serious problems.^63^,^64^ Isolated cases of ototoxicity, alternation of taste (metallic or loss of taste), visual hallucination and visual disturbances (such as conjunctivitis, episcleritis and uveitis) has been reported after therapy with pamidronate,^50^,^63^,^64^ but these reactions were transient and reversible after discontinuation of intravenous BPs.^3^,^50^

## Conclusions

Oral bisphosphonates generally well accepted by postmenopausal women, but their tolerability and long term use is complicated. Persistence with daily oral bisphosphonates for long time are shown to be suboptimal in many patients, because of the gastrointestinal problem and posture requirements, leading to an increased fracture incidence in non-compliant patients.^6^ This negatively impacts treatment outcomes, leading to a reduced clinical benefit, thus there is a need to improve overall adherence for bisphosphonate treatment in order to achieve maximum treatment effects. To increase bone mineral density, reduce risk of fracture and maximise tolerability, the future of pharmacologic treatments for osteoporosis will likely decrease the frequency of administration with use of more potent intravenous formulations and more prolong inhibition of bone resorption.^65^

Until recently, intravenous BPs was only used to treat metastatic bone disorder or complication of malignancies such as hypercalcemia or bone pain. Pamidronate could be used in a variety of conditions associated with low bone mass but due to lack of FDA approval and the requirement of a long infusion time is only limited as an off-label indication for treatment of a group of postmenopausal women with osteoporosis.^15^

New, more powerful third generation BPs can be administered more rapidly with more prolong effect on bone resorption. Currently, zoledronic acid at the dosage of 5 mg annually and ibandronate at the dosage of 3 mg every 3 months are parenteral bisphosphonate that have received FDA approval for the treatment of postmenopausal osteoporosis.^15^ (Table 1) These products are injected in few minutes, a great advantage compared to the 4 or more hours infusion required for pamidronate. Although these intravenous formulations are cost more than oral ones but in spite of efficacy, compliance and acceptance of postmenopausal women for prolong use seems becoming more popular in next few years.^68^
